# DNA stretching on the wall surfaces in curved microchannels with different radii

**DOI:** 10.1186/1556-276X-9-382

**Published:** 2014-08-07

**Authors:** Shou-Shing Hsieh, Fong-He Wu, Ming-Ju Tsai

**Affiliations:** 1 Department of Mechanical and Electromechanical Engineering, National Sun Yat-Sen University, 80424 Kaohsiung, Taiwan

**Keywords:** DNA stretching, Microchannel, μPIV, Curve effect

## Abstract

DNA molecule conformation dynamics and stretching were made on semi-circular surfaces with different radii (500 to 5,000 μm) in microchannels measuring 200 μm × 200 μm in cross section. Five different buffer solutions - 1× Tris-acetate-EDTA (TAE), 1× Tris-borate-EDTA (TBE), 1× Tris-EDTA (TE), 1× Tris-phosphate-EDTA (TPE), and 1× Tris-buffered saline (TBS) solutions - were used with a variety of viscosity such as 40, 60, and 80 cP, with resultant 10^−4^ ≤ Re ≤ 10^−3^ and the corresponding 5 ≤ Wi ≤ 12. The test fluids were seeded with JOJO-1 tracer particles for flow visualization and driven through the test channels via a piezoelectric (PZT) micropump. Micro particle image velocimetry (μPIV) measuring technique was applied for the centered-plane velocity distribution measurements. It is found that the radius effect on the stretch ratio of DNA dependence is significant. The stretch ratio becomes larger as the radius becomes small due to the larger centrifugal force. Consequently, the maximum stretch was found at the center of the channel with a radius of 500 μm.

## Background

Fully stretched DNA molecules are very important with regard to advancing the genomic sciences and analyses in order to understand the physical and biological properties of DNA, including the ability to directly manipulate and visualize single DNA molecules. In fact, engineering DNA stretching would be a key step in the development of the next generation of biological microfluidic devices [1,2].

Microfluidics is the study of behavior manipulation and control of fluids confined to micrometer dimensions, typically 1 to 100 μm. Transport in the microchannels is the major phenomenon; it includes flow detections, liquid transport, control of molecular transport like DNA molecule conformation dynamics, measurement of bulk-level rheological properties, and separation techniques with biophysical and genomic applications because they generate defined fluid flows that manipulate large DNA molecules [3]. In addition, understanding the complex behavior of DNA molecules flowing in microchannels is essential to the realization of lap-on-a-chip (LOC) and micro total analysis system (μTAS) intended to systematically manipulate, process, and analyze these molecules. The presence of DNA molecules gives the fluid viscoelastic behavior that may change the base flow pattern in curved channels [4].

Two general approaches to DNA stretching are in common use: DNA is stretched in a solution as it flows through a microchannel, or it is stretched on a solid surface. For the latter, the conditions required for significant DNA stretching include high shear rates and high pressure gradient operations with a pressure-driven flow, due to non-slip boundary conditions on the wall. The shear flow existing at the channel walls could stretch DNA molecules. The degree of stretching is correlated with the Weissenberg number of the flow, Wi = *τ*_,_ where *τ* is a characteristic relaxation time for the molecule in the solution and is a characteristic shear rate based on the flow in the channel. For the past two decades, DNA molecules have served as a model system for single molecule semi-flexible polymer (larger persistence length of approximately 65 mm) dynamics and can be fluorescently labeled for direct observation with videomicroscopy [5], revealing a DNA solution non-equilibrium microstructure, DNA- solvent interactions, and DNA macromolecular transport phenomena. Moreover, increased interactions between DNA molecules and channel surfaces result in non-Newtonian flow behavior, even in a dilute DNA molecule solution.

When the laminar flow passes through the curved channels/ducts, the centrifugal force pushes the fluid from the center of the channel when the bulk fluid flows with high velocity to the outer side, while the fluid at the outer wall is pressed either upwards or downwards, thus producing two vortices to fill the entire channel at a cross section along the downstream [6]. The mean flow velocity and the curvature of the channel can determine the centrifugal force, which is governed by an important dimensionless parameter of Dean number (Dn = Re (*d*_h_/*R*)^0.5^), including the flow Reynolds number (Re) and the duct hydraulic diameter (*d*_h_) to the curvature of the channel/duct (*R*). Here, the Reynolds number is defined as Re=ρUdh/μγ˙, where *ρ* is the solution density, *U* is the average velocity, *μ* is the solution viscosity, and $\left(\dot{\gamma }\right)$ is the solution shear rate.

Research on shear flow [3,7,8] has been conducted in order to model the conformation of DNA molecules for an extended time. These studies reported that the stretching of the DNA molecules subject to shear flow is a function of Wi and *τ* in the flow. In this study, λDNA molecules were stretched on curved wall surfaces in different curved ducts in pressure-driven flows and visualized as well as measured via micro particle image velocimetry (μPIV) and an optical system. Special attention will be paid to examining the effect of different radii of the curved duct (i.e., Dn), buffer solutions, and the viscosity of the solution. Moreover, viscoelastic (i.e., non-Newtonian) flow in dilute DNA solution will also be examined.

## Methods

### PDMS flow cell fabrication

**Figure 1:**
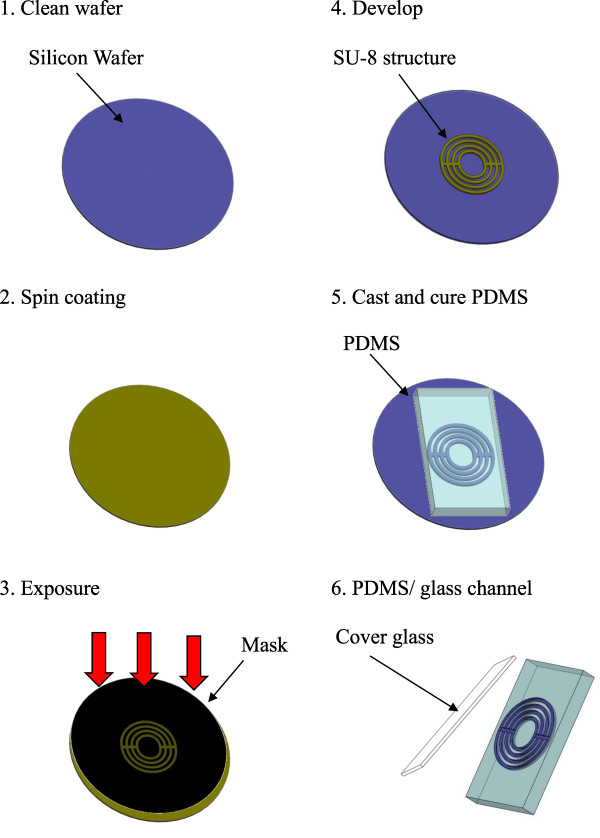
Fabrication of the present curved channel.

In this study, we used a 200 μm × 200 μm microchannel, as shown in Figure 1. The polydimethylsiloxane (PDMS) channels were fabricated in-house at the University Microsystem Laboratory by casting open ten concentric circular-slot channels from PDMS and sealing it with the same material. At the center of these ten concentric circles, an up/down plenum was drilled to allow the buffer solution and DNA molecules to flow in/out; thus, the circular ducts became two symmetric half semi-circle ducts. The casting mold was made by SU-8 deep UV lithography. The detailed SU-8 mold design and PDMS curved channel fabrication created through UV lithography can be found in [1], with slight modifications for the photomask. Table 1 lists the fabrication parameters of the present curved microchannels.

**Table 1:** Fabrication parameters of curved microchannel

	**Relevant parameter**
SU-8 fabrication processes	
Spin coating	550 rpm (80 s)
	800 rpm (70 s)
Soft bake	65°C (3 min)
	95°C (21 min)
	65°C (3 min)
	Room temperature (30 min)
Exposure	Total time 30 s
Post exposure bake	65°C (3 min)
	95°C (12 min)
	65°C (3 min)
Hard bake	95°C (3 min)
Remarks	Substrate type: silicon wafer
	Photoresist: SU8-2100 (MicroChem)
	Depth: 200 μm
	Photomask: film mask
	(FUJI HPB-S 7mil, 20,000 DPI)
PDMS fabrication processes	
PDMS prepared	10:1 Sylgard-184 A/Sylgard-184 B mixture
Bake	70°C (21 min)

For the tested channels, precise information on their dimensions is extremely important to obtain an accurate evaluation of this microchannel. The depth, width, and length were measured optically within an accuracy of ±0.2%. The surface roughness of the channel was measured with a surface profilometer. During the experiments, the surface of the flow channel was so designed that the surface was kept hydrophilic in order to have the buffer solution flow through the microchannels with a definite surface resistance. Pressure gradients in the present curved channels generated modified (due to centrifugal force) parabolic flow, such that shear flow occurred near the channel walls. Furthermore, microfluidic semi-circular curved ducts created a periodic oscillating flow, in which flow pressure gradient alternated directions at a definite time and extended observations of DNA molecules.

### DNA visualization and buffer solution preparation

**Figure 2:**
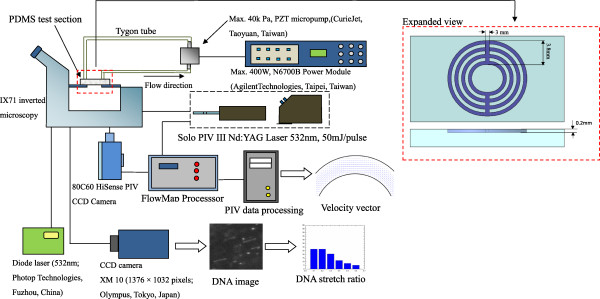
Schematic for the present measuring instruments.

An experimental setup scheme combined with a laser light source (Ar-ion laser 488 nm/HeNe laser 532 nm) and scanning system used to implement μPIV measurement is shown in Figure 2. The flow cell was mounted onto an epifluorescent microscope (IX71/FV300, Olympus, Tokyo, Japan) equipped with a × 40 magnification and NA 0.85 air immersion objective lens, following the description in [2,9]. The use of the μPIV technique is very attractive in microfluidics because it helps to determine the detailed flow phenomena of microsystems by utilizing flow-tracing particles to map the flow in the microchannels. Streak images and video microscopy assist in the investigation into the flow kinematics in the circular curved microchannels; μPIV is used to quantify the flow field in the vicinity of the curved channels. In this study, the stained DNA molecules (JOJO-1, Invitrogen, Carlsbad, CA, USA) were used as seeding. The probe used to visualize the DNA was JOJO-1 at a dye with base pair ratio of 1:5. Incubation for the DNA and probe was initiated. The dyed λDNA had a contour length (*L*_e_) of 21 μm and the longest relaxation time (*τ*) of 7.6 s.

### Shear flow system

A custom-made flow system was developed to enable the simultaneous generation of controlled shear flows and visualization of the DNA molecular conformation dynamics. The present DNA solution was found to be highly shear thinning at high shear rates, with a shear viscosity *μ* (cP) defined as the power law $\mu =\;77{\dot{\gamma }}^{-0.7}$ (one of typical relations). Flows of water and diluted DNA solution (λDNA, 31.5 × 10^6^ D, 48,502 base pairs) are explored in the microchannels. The DNA solution was diluted to a concentration of 0.0325 μg/ml using five different buffers (see Tables 2 and 3 for details). In order to increase the viscous drag, the viscosity of the buffer solution was adjusted from 40 to 80 cP by adding a proper amount of sucrose. The test fluids, as stated previously, were seeded with JOJO-1 tracer particles for flow visualization and driven through the circular curved ducts using a piezoelectric (PZT) micropump. A microfilter was placed between the pressure regulator and the flow meter to eliminate any particles (>0.1 μm) or bubbles (>0.1 μm). A tracing particle of stained DNA molecules was used for μPIV measurements between the flow meter and the inlet and outlet of the channel. The mass flow rate was estimated through a stopwatch to count how long the buffer solution took to complete a flow loop, and the total weight of the buffer solution in a flow loop was measured by a microbalance. The mass flow rate found in this study was about 3 × 10^−4^ to 6 × 10^−4^ ml/min. The errors of the flow rate measurement were estimated to be less than ±3%. The DNA solution was delivered into the circular duct with two equal flow rate fluid delivery lines, with a very small Reynolds number in the range of 0.326 × 10^−3^ to 1.87 × 10^−3^, in which molecular diffusion was a major mechanism for mixing. The Reynolds number was based on the shear rate-dependent viscosity *μ*, as stated previously. The characteristic shear rate $\dot{\gamma }$ used for calculating Wi was taken to be the average velocity *U* divided by the channel half width *w*/2.

**Table 2:** Buffer solution used in the study

	**1× TE**			**1× TAE**			**1× TBE**			**1× TPE**			**1× TBS**		
Viscosity (cP)	40	60	80	40	60	80	40	60	80	40	60	80	40	60	80
Sucrose (g/ml)	1.437	1.606	1.726	1.437	1.606	1.726	1.437	1.606	1.726	1.437	1.606	1.726	1.437	1.606	1.726
Tris base concentration (mM)	10			40			90			90			50		
EDTA concentration (mM)	1			1			2			2			None		
Other ion concentration	5.2 mM of hydrochloric acid			20 mM of acetic acid			90 mM of boric acid			26 mM of phosphoric acid			150 mM of sodium chloride		
pH	8			8			8			8			8		
Lambda DNA (μg/ml)	0.0325														
JOJO-1 concentration (mM)	0.02														

**Table 3:** Relevant parameters of the flow under study

**Parameter**	**Value**		
Pressure drop	34 Pa, 44 Pa, 57 Pa		
Power consumption	0.06 W, 0.068 W, 0.08 W		
DNA molecular concentration	0.0325 μg/ml		
Working fluid viscosity, *μ* (cP)	40	60	80
Reynolds number, Re (×10^−3^)	1.2 to 1.87	0.561 to 0.828	0.326 to 0.486
Dean number (×10^−4^)	1.7 to 8.4	0.8 to 4.1	0.4 to 2.4
Relaxation time, *τ*_R_ (Rouse model)	4.2	6.31	8.41
Relaxation time, *τ*_Z_ (Zimm model)	3.1	4.6	6.1
Relaxation time, *τ* (present study)	3.82	5.6	7.6
Weissenberg number, Wi	6.7 to 11	7.2 to 11.3	8 to 12

### μPIV system

The μPIV utilizes flow-tracing particles (stained DNA molecules) to map the flow in the microchannels. The setup shown in Figure 2 was based on two-pulsed Nd:YAG lasers (New Wave SoloII, 30 mJ, double cavity; New Wave Research, Inc., Fremont, CA, USA) firing on the second harmonic (green 532 nm). A detailed description of the μPIV setup can be found in [9]. The concentration of the stained DNA molecules, based on the interrogation volume, was less than 8 × 10^7^ particles/ml. The images were recorded using a Dantec 80C77 Hisense PIV 1,344 × 1,024 × 12 bit interface transfer camera (Dantec Dynamics A/S, Skovlunde, Denmark). A total of five images were taken for each flow field with a spatial resolution of 64 × 64 pixels. The interrogation cell overlay was 50%. The background noise effect was removed by subtracting the background intensity from captured images. In addition, an ensemble averaging 20 images consecutively captured in 4 s was used to obtain the velocity measurements and to avoid the Brownian motion of the stained DNA molecules. A total of 800 sets of data were taken at each location for a specified Re. The selection of 800 datasets was based on the examination of the data convergence. Each measurement was repeated at least five times under specific conditions.

## Results and discussion

**Figure 3:**
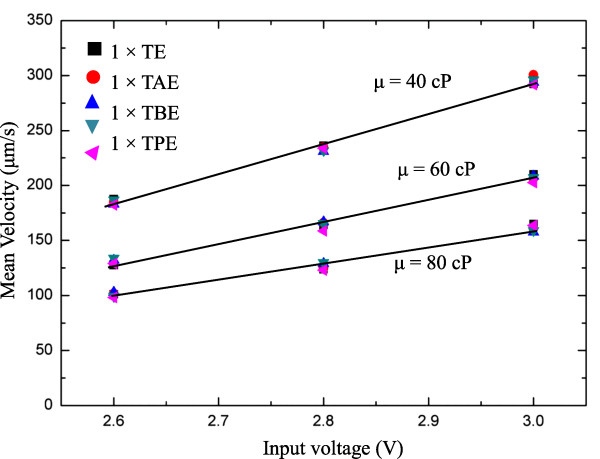
Input voltage (DC) vs velocity for the present piezoelectric (PZT) micropump.

Prior to the formal runs, the velocity in different buffer solutions with varied viscosity for the present PZT pump should first be calibrated. Through μPIV measurements, average velocity for five different buffers with three different viscosities of 40, 60, and 80 cP was measured and calculated. The results are now plotted against the PZT input voltage, as shown in Figure 3. Generally, the distribution showed a common trend in which a linear proportionality was present. The higher viscosity caused a lower velocity distribution, as expected. The slope of the distribution became smaller as the viscosity increased. The velocity magnitude spans from 100 to 300 μm/s as the input voltage rises from 2.6 to 3.0 V (direct current (DC)). The buffer solution effect on the velocity seems not to have been noted.

**Figure 4:**
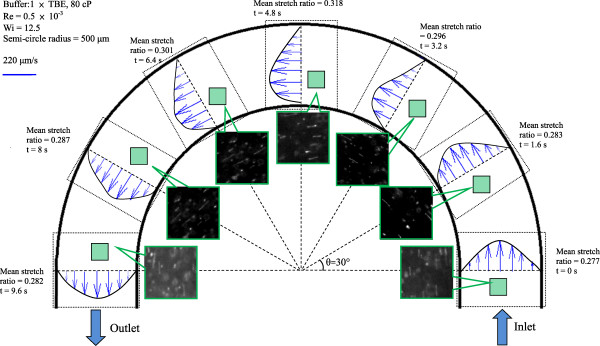
**Flow characteristic of the present curved channel for a typical case (*R*** **= 500 μm).**

There are ten semi-circular channels with different radii from 500 to 5,000 μm. With different curvature effects (i.e., different Dean numbers), the stretching effect differs. It was found that due to the higher Dn, the smaller the radius, the longer the stretching. Therefore, only data for the radius of 500 μm with 1× Tris-borate-EDTA (TBE) and 80 cP at Re = 5 × 10^−4^ (Wi = 12.5) was presented, as shown in Figure 4. Seven sequent images of the present stretching were illustrated with different stretching ratios at the corresponding time. A total period of a cycle takes about 9.6 s with each time interval of 1.6 s. The maximum stretch occurred at the center of the semi-circular duct. The stretch ratio was oscillatory rather than monotonic due to the pressure recovery when the flow moved though the curved channels. An accompanying plot of the local velocity distribution for each stretch was also provided to depict the local velocity gradient. This also explains why the maximum stretch occurred at the center of the semi-circle, and the oscillating behavior began. As stated previously, the local velocity fields developed via μPIV can be used to quantify the magnitude of the flow around the semi-circular duct, as well as the strength of the shear force. In each image, the DNA molecule stretch was clearly observed as the corresponding stretch ratio increases, confirming cycling between stretched (0 ≤ *θ* ≤ 90°) and relaxed (90° < *θ* ≤ 180°) forms. Due to the parabolic velocity profile, the DNA stretch was not uniform across the microchannel and DNA molecules near inner walls were more stretched than those occupying the central portion and outer wall of the channel due to the centrifugal force.

**Figure 5:**
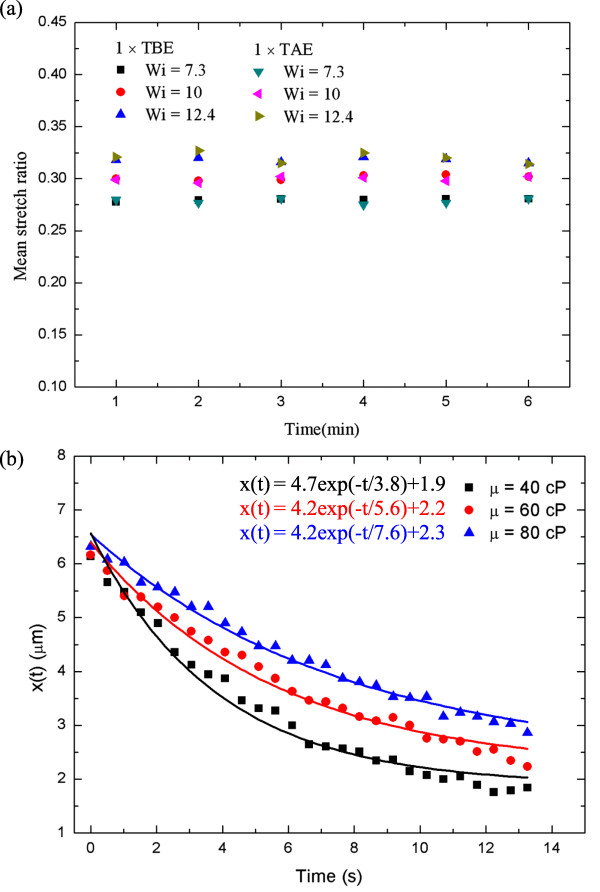
**DNA stretching and DNA molecule elongation. (a)** Time history of DNA stretching at different Wi. **(b)** DNA molecule elongation length vs time.

Figure 5a shows the mean stretch ratio distribution versus time in two different buffer solutions with different Wi (7.3 to 12.4). As expected, the buffer solution seems to exhibit no significant influence on the stretch ratio; it increases as the Wi increases. In addition, the mean stretch seems constant and is independent of time in a time period of 6 min. DNA molecule elongation was plotted against time and is shown in Figure 5b, in which an exponential decay form was found for three different viscosities: 40, 60, and 80 cP. The longest elongation was secured with a viscosity of 80 cP, as expected, while the shortest is for 40 cP. Taking a close-up look, one may find different relaxation times of 3.8, 5.6, and 7.6 s for different viscosities of 40, 60, and 80 cP, respectively. With time passing, elongation of the DNA molecules reaches a minimum for each viscosity which has a value of 1.9, 2.2, and 2.3 μm for the corresponding viscosities of 40, 60, and 80 cP at a time of about 13 s.

**Figure 6:**
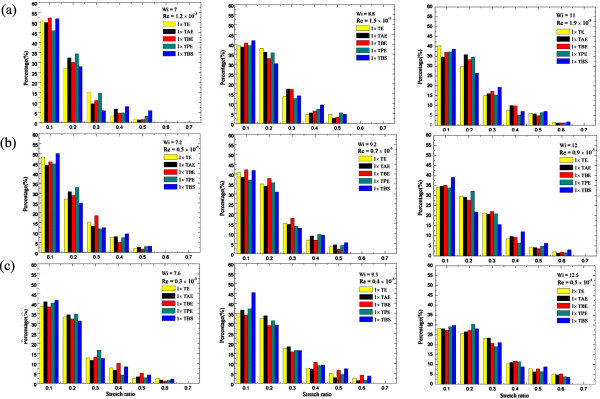
**Stretching ratio histogram for different buffers with different viscosities. (a)** 40 cP, **(b)** 60 cP, and **(c)** 80 cP.

**Figure 7:**
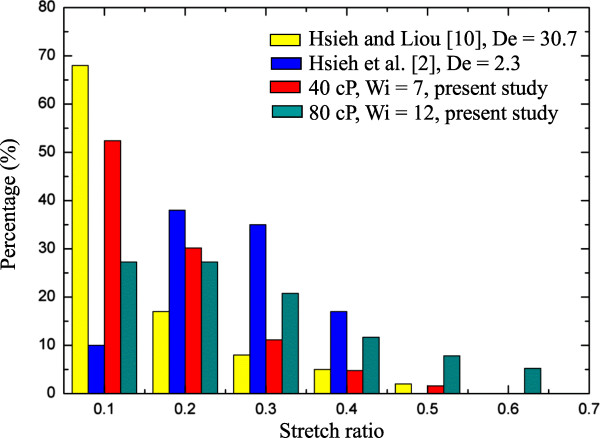
Comparisons with the related previous studies for DNA stretching.

Figure 6a,b,c depicts the DNA molecule stretch ratio histogram for all five different buffers with three viscosities, respectively, for Wi (Re) from 7.6 (0.3 × 10^−3^) to 12.5 (0.5 × 10^−3^). Generally, buffer dependence again seems not to have been noted; furthermore, most DNA molecules (about two thirds) are in the range of stretch ratio less than 0.2 regardless of the buffers and viscosity, although this value (0.2) would increase as the viscosity increases. For instance, with the highest viscosity of 80 cP, there were about 5% of DNA molecules in which the stretch ratio could reach to 0.65. Common features for each among these three different viscosities can be seen; it was found that the extension was positive, and the minimum stretch ratio was approximate 0.1 of 40% to 45% of the DNA molecules. The stretch ratio would increase to 0.65 as the Wi ≥ 11 for viscosity of 40 and 60 cP, as shown in Figure 6a,b; for the viscosity of 80 cP, this happens when Wi ≥ 7.6, which can be seen in Figure 6c. In addition, more than 5% of the DNA molecules can reach this value (i.e., stretch ratio 0.65) for Wi = 12.5. Comparisons were also made, as shown in Figure 7, with those related studies for the viscosities of 40 and 80 cP. The present data are consistently higher than those of previous studies [2,10] with regard to both the percentage of the stretched DNA molecules and their stretch ratio. In fact, about 10% of DNA molecule stretch can reach the ratio of 0.52, and about 7% of DNA molecules can reach 0.63. Again, these are higher levels than those of previous studies. Table 4 shows a summary of the DNA mean stretching rate for all the cases under study.

**Table 4:** DNA mean stretching rate

**Input voltage (DC)**	**Buffer viscosity (cP)**	**1× TE**	**1× TAE**	**1× TBE**	**1× TPE**	**1× TBS**
2.6 V	40	0.26	0.252	0.253	0.265	0.262
	60	0.271	0.266	0.271	0.2676	0.2754
	80	0.278	0.283	0.281	0.28	0.2844
2.8 V	40	0.284	0.2867	0.283	0.2867	0.2922
	60	0.288	0.293	0.289	0.2917	0.2953
	80	0.311	0.301	0.3	0.3035	0.308
3.0 V	40	0.302	0.309	0.302	0.3031	0.3061
	60	0.317	0.315	0.307	0.316	0.315
	80	0.318	0.317	0.318	0.3165	0.317

**Figure 8:**
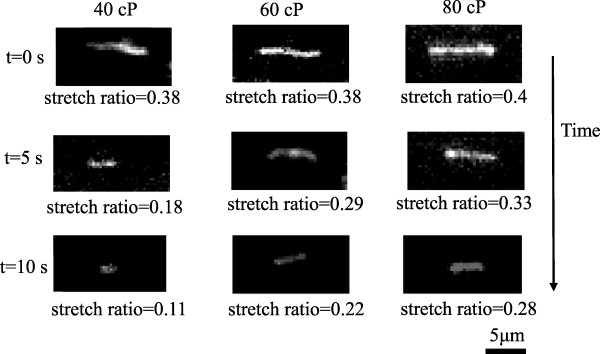
**Representative of DNA recoiling at different times (Δ*t*** **= 5 s) for 1× TBE.**

**Figure 9:**
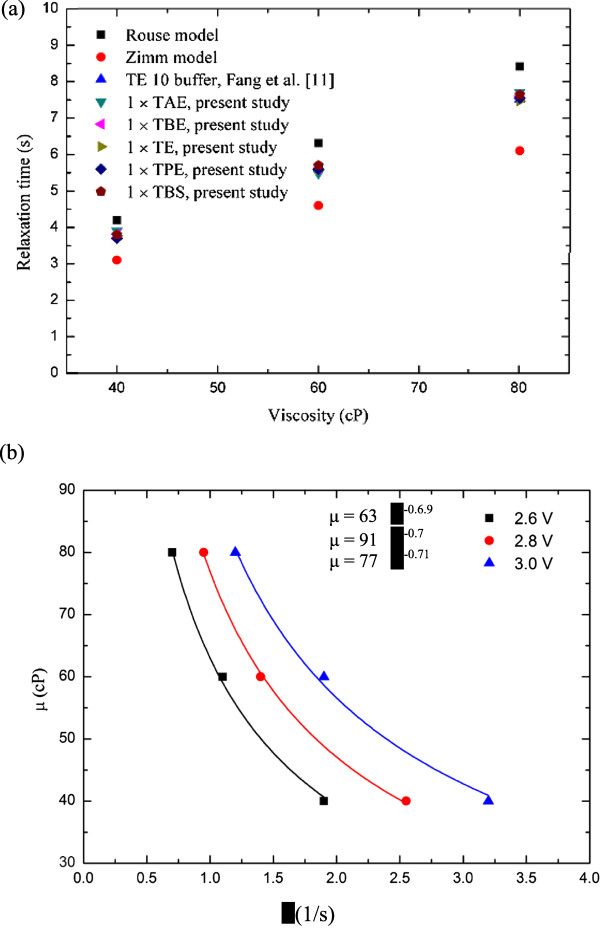
**Graphs showing (a) relaxation time vs viscosity and (b) μ vs**$\dot{\gamma }$**.**

**Figure 10:**
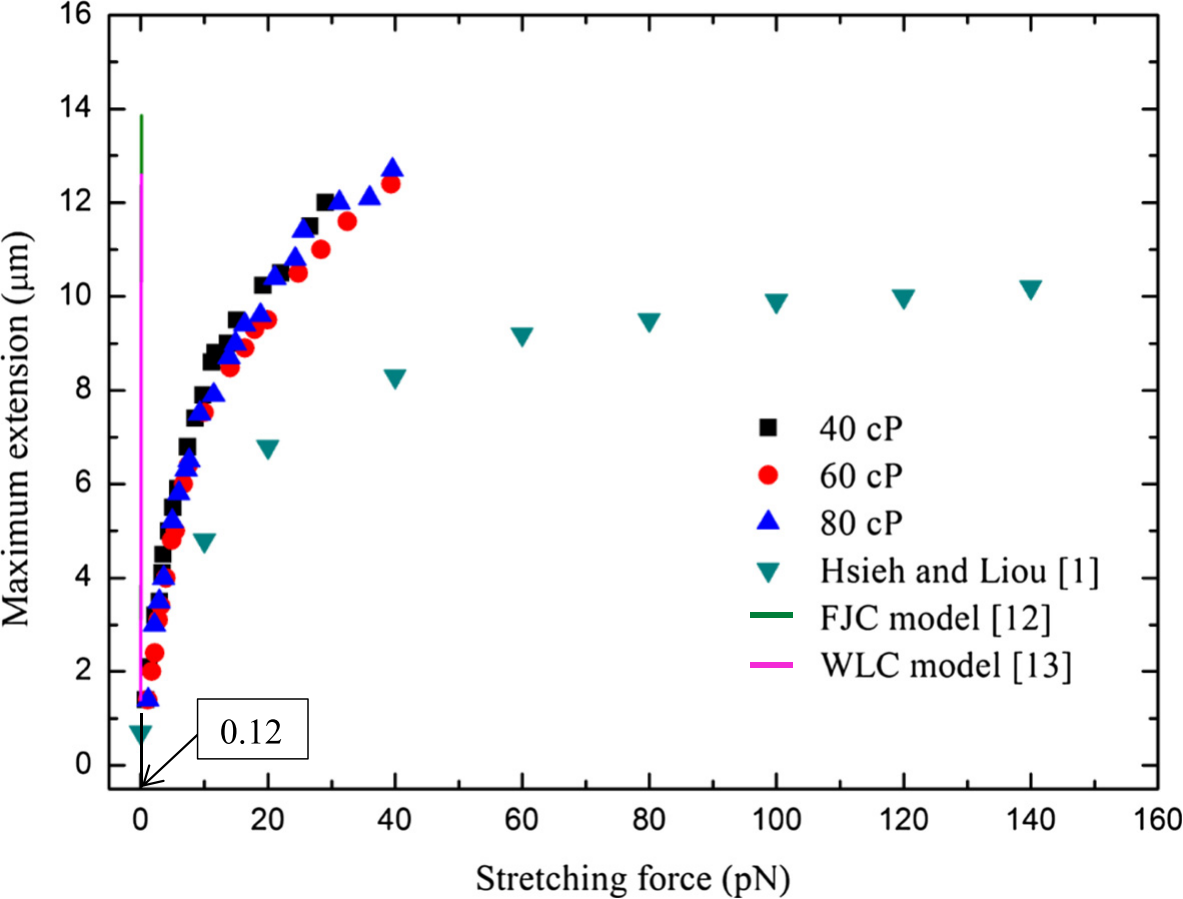
Comparisons with those of previous studies.

Based on the DNA molecule conformation history, it was found that the entire semi-annular duct exhibited two different opposite trends. First, in the first half duct (i.e., *θ* ≤ 90°), the DNA molecules obviously experienced stretching; however, for the second half duct (i.e., 90° < *θ* ≤ 180°), it experienced an opposite behavior like recoiling. This is also evidenced by Figure 8, as time increases with an interval of Δ*t* = 5 s. Figure 9a,b shows the relaxation time versus viscosity and the functional relationship of viscosity with $\dot{\gamma }$, respectively. Following Figure 9a, one may conclude that the relaxation time was a function of $\dot{\gamma }$ as well. Also included in Figure 9a are those from the Rouse/Zimm model and Fang et al. [11] for comparison. Good agreement and consistency were found. In fact, the present results for the five different buffers under study were between those of existing models. In Figure 9b, the viscosity which was correlated in terms of power law with an average power of 0.7 was found under different DC voltage inputs. The maximum stretch of the stretching force was plotted and is shown in Figure 9a with comparisons to those of listed models [12,13]. The data shown strongly indicated that a small stretching force was needed, as compared to the existing model with the same stretching length. However, the developing trend of the present study is the same as those of existing models [12]. The viscosity effect for *μ* = 40 ~ 80 cP of the present study seems not to have been noted as far as the stretching force is concerned, as shown in Figure 10. The Freely Jointed chin model (FJC) and Wormlike chain model (WLC) cannot be compared due to their small values (approximately 0.12 pN). The stretch length of the present study is clearly quadratically dependent on the stretching force. Due to the fact that both FJC [12] and WLC [13] models may not be applicable, Hsieh and Liu's results [1] were now included in Figure 10 for comparison. It can be seen that less stretching force was needed for the present study compared to those of Hsieh and Liu [1].

**Figure 11:**
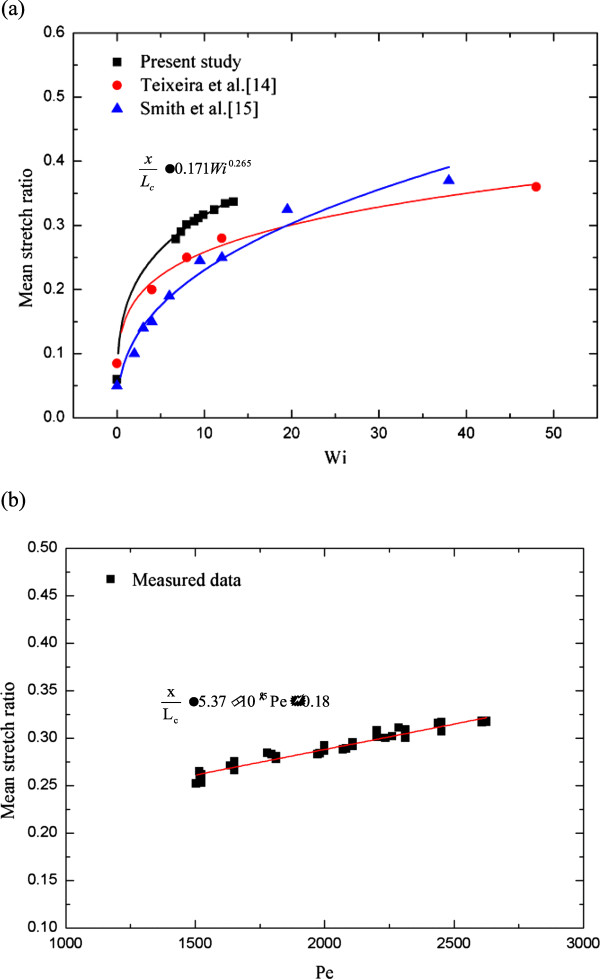
Graphs showing (a) mean stretch ratio vs Wi and (b) mean stretch ratio vs Pe.

Finally, data for mean stretch ratio were correlated in a power law from of Wi as *x*/*L*_c_ = 0.17 Wi^0.265^, as indicated in Figure 11a. Teixeira et al.'s [14] and Smith et al.'s [15] results were also included in Figure 11a. Again, the present results show a large stretch with a definite Wi. Another correlation of mean stretch ratio as a function of Pe is shown in Figure 11b. A straight line relation was found in the form of *x*/*L*_c_ = 5.37 × 10^−5^ Pe + 0.18, and the initial stretch length was obtained as Pe equals zero in this study.

## Conclusions

DNA molecule dynamics in curved (semi-circle, 0° ≤ *θ* ≤ 180°) microchannels with different radii for five different buffer solutions of 1× Tris-acetate-EDTA (TAE), 1× Tris-borate-EDTA (TBE), 1× Tris-EDTA (TE), 1× Tris-phosphate-EDTA (TPE), and 1× Tris-buffered saline (TBS) with a variety of viscosity such as 40, 60, and 80 cP were extensively studied for 10^−4^ ≤ Re ≤10^−3^ and 5 ≤ Wi ≤12. The major findings drawn are as follows:

1. Radius effect was significantly noted with maximum stretch ratio occurring at the center of the semi-circle (*θ* = 90°) with a radius of 500 μm.

2. The oscillatory/recovery nature of the present stretching behavior was found.

3. The buffer solution type seems to have no significant influence on the stretch ratio, with no viscosity effect.

4. The correlation of *x*/*L*_c_ was developed for parameters of Wi and Pe, respectively, with different functional relationships.

## Abbreviations

LOC: lap-on-a-chip; PZT: piezoelectric; μTAS: micro total analysis system.

## Competing interests

The authors declare that they have no competing interests.

## Authors’ contributions

SSH provided the idea and drafted the manuscript. FHW was responsible for carrying out the experimental work and the basic result analysis, and designed the experiment. MJT assisted with the result analysis and paperwork. All authors read and approved the final manuscript.

## Authors’ information

SSH is a professor at the Department of Mechanical and Electro Mechanical Engineering, National Sun Yat-Sen University, Kaohsiung, Taiwan, Republic of China. FHW is a student working towards a master's degree at the Department of Mechanical and Electro Mechanical Engineering, National Sun Yat-Sen University, Kaohsiung, Taiwan, Republic of China. MJT is a student working towards a master's degree at the Department of Mechanical and Electro Mechanical Engineering, National Sun Yat-Sen University, Kaohsiung, Taiwan, Republic of China.
